# Maternal imprinting and determinants of neonates’ immune function in the SEPAGES mother-child cohort

**DOI:** 10.3389/fimmu.2023.1136749

**Published:** 2023-04-04

**Authors:** Olivier Manches, Khémary Um, Anne Boudier, Yasmina Maddouri, Sarah Lyon-Caen, Sam Bayat, Rémy Slama, Claire Philippat, Valérie Siroux, Laurence Chaperot

**Affiliations:** ^1^ EFS, Recherche et Développement, Grenoble, France; ^2^ Université Grenoble-Alpes, INSERM U1209, CNRS UMR, Institute for Advanced Biosciences, Grenoble, France; ^3^ Department of Pulmonology and Physiology, CHU Grenoble-Alpes, Grenoble, France

**Keywords:** neonate, immune system, pregnancy, intrauterine immune imprinting, cytokines

## Abstract

**Introduction:**

Immune function in pregnancy is influenced by host-specific and environmental factors. This may impact fetal immune development, but the link between maternal and neonatal immune function is still poorly characterized. Here, we investigate the relationship between maternal and neonatal immune function, and identify factors affecting the association between maternal and child cytokine secretion.

**Methods:**

In the French prospective cohort SEPAGES, blood samples were obtained from pregnant women (n=322) at gestational week 20 ± 4 and from their child at birth (n=156). Maternal and cord blood cytokine and chemokine (CK) levels were measured at baseline in all subjects and after T cell or dendritic cell activation with phytohemagglutinin or R848 (in total 29 and 27 measures in maternal and cord blood samples, respectively). Associations between environmental, individual factors and CK level were estimated by linear regression modeling. The maternal-cord blood CK relations were assessed by Pearson correlation and regression models.

**Results:**

We observed that pregnant women and neonates displayed specific CK secretion profiles in the innate and adaptive compartments at baseline and upon activation. Activation of T cells in cord blood induced high levels of IL-2, but low levels of IFNγ, IL-13 or IL-10, in comparison to maternal blood samples. Elsewhere, neonatal innate immune responses were characterized by low production of IFNα, while productions of IL-1β, IL-6, IL-8, IL-10 and TNFα were higher than maternal responses. Strong correlations were observed between most CK after activation in maternal and cord blood samples. Strikingly, a statistical association between global mother and child cytokine profiles was evidenced. Correlations were observed between some individual CK of pregnant women and their children, both at baseline (MCP1, RANTES) and after activation with R848 (IL-6, IL-8 and IL-10). We looked for factors which could influence cytokine secretion in maternal or cord blood, and found that leucocyte counts, maternal age, pre-conception BMI, smoking and season were associated with the levels of several CK in mothers or children.

**Discussion:**

Our study reveals *in utero* immune imprinting influencing immune responses in infants, opening the way to investigate the mechanisms responsible for this imprinting. Whether such influences have long lasting effects on children health warrants further investigation.

## 1 Introduction

Immune dysregulation plays a central role in the pathogenesis of many diseases. Immune functions are continuously influenced by host and environmental factors over the course of life. Age and gender greatly influence the immune system in healthy individuals, which may participate in differential disease susceptibility (1, 2). In addition, season (1, 2) and past exposure to pathogens and commensal microbes (3) have been shown to affect the human immune system. During pregnancy, considerable changes in the maternal immune system occur, allowing tolerance to the fetal allograft while preserving protection against pathogens (4, 5). The maternal immune profile can also be influenced by maternal factors [Body-Mass Index (BMI), obesity (5–8), diabetes (9)] or environmental factors [smoking, air pollution, nutrition, or infections (10–13)]. All these factors may affect *in utero* fetal immune development, and drive long lasting changes to individual immunity. Understanding the feto-maternal immunological dialogue may help identify mechanisms influencing child and adult immune function and health.

The newborn immune system, still poorly understood, is highly different from children, adolescents or adults, with both qualitative and quantitative differences in many aspects of immunity (14, 15). It is highly adapted to early life particular challenges that imply tolerating commensal microbes while fighting pathogens. Newborn innate compartment of immunity is hyperactive, and trained immunity is emerging as an important component of early life defense against pathogens. Elsewhere, increased susceptibility to infection in neonates might relate to development of disease tolerance, involving active immunosuppression processes, as a defense strategy. Several studies have shown that adaptive immunity, and specially T cells from healthy neonates are functionally immature, with very low Th1 and Th2 cytokines but high IL-2 producing abilities (16). Relationships between maternal and fetal helper T-cell responses were mostly described in studies that aimed at understanding how maternal allergy confers an allergy risk to the offspring. Upon stimulation with phytohemagglutinin (PHA) or Staphylococcal enterotoxin B in cohorts comprising high proportions of allergic mothers, correlations (mostly positive) were observed between maternal and neonate or child production of IFNγ, IL-10, IL-4 or IL-13 (17–19), suggesting an *in utero* immune imprinting of adaptive immunity. Very recently, a positive association between maternal prenatal IFNγ:IL-13 ratio and neonatal LPS-induced IL-6 production was described (20). While some maternal cells can traverse the placental barrier, establishing microchimerism in children (21), little is known on how maternal cytokines can influence offspring development (22). In the context of SARS-CoV-2 infection during pregnancy, it has been shown that inflammatory responses at the maternal–fetal interface were associated with the presence of IL-8 in the neonatal circulation (13); moreover, neonates born from infected mothers had profound alterations of circulating innate immune cells and cytokines (23). In the context of maternal infection by HBV, innate immune cells of neonates displayed enhanced maturation status, and plasma IL-12p40 and IFNα2 were elevated while IL-10 was low (24). These results suggest a potential imprinting of immune responses during fetal development induced by maternal infection and inflammation, in the absence of vertical virus transmission.

Demonstrating *in utero* immune imprinting of the fetal immune system is still a challenge, and whether such imprinting can influence immunity in children and hence impact the health of the individual is unclear and poorly studied in healthy population-based cohorts. In particular, to our knowledge, no large mother-child population cohort has yet deeply characterized the immune state and innate immune function in healthy mothers and their neonates.

The main objective of our study was to investigate the relationship between maternal and umbilical cord blood systemic circulating cytokine levels at baseline, and after T cell or dendritic cell activation in whole blood. The second objective was to identify the factors affecting maternal and children cytokine levels.

## 2 Materials and methods

### 2.1 Study population

This study is based on the data from the French mother-child SEPAGES (Assessment of Air Pollution exposure during Pregnancy and Effect on Health) cohort, aimed at characterizing the effect of maternal and child exposure to environmental pollutants on health. As extensively described by Lyon-Caen et al. (25), 484 pregnant women were recruited from July 2014 to July 2017 in eight obstetrical ultrasonography practices located in the Grenoble area in the French Alps. To be included women had to be pregnant by less than 19 gestational weeks, be older than 18 years old, to have a singleton pregnancy, to be planning to give birth in one of the four maternities clinics from Grenoble area and to live in the study area. Questionnaires, interviews and clinical examinations during and after pregnancy were used to collect sociodemographic, environmental and medical information on the children and their parents. This study included 322 pregnant women with plasma samples collected at gestational week 18-26 (mid gestation) and 156 newborns with cord blood samples collected at delivery (see flow chart of the study, **Supplementary Figure 1**). All mothers and fathers signed an informed consent form for themselves and their child. Ethical agreements were obtained from the Commission Nationale de l’Informatique et des Libertés (CNIL, the French data privacy institution, authorization n°2014-263, 2014-06-26),the CPP (Comité de Protection des Personnes Sud-Est V, authorization n°13-CHUG-44, 2014-02-25) and the Agence nationale de sécurité du médicament et des produits de santé (ANSM, authorization n°2013-A01491-44, 2013-12-17).

### 2.2 Biospecimen collection

Non-fasting maternal blood was collected by trained SEPAGES field workers during a study visit at the participants’ homes. Cord blood was collected at birth by midwifes. Blood samples were collected in BD Medical 368886 vacutainer tube (lithium heparin) for immunological analyses (cell culture and plasma separation), and in BD Medical 368861 vacutainer tube (EDTA) for cell counting.

After collection, samples were transported on ice to the EFS laboratory, they were put on a rotated device at least for 5 minutes before experiments, in order to ensure homogeneous cell content. Most blood samples were handled within 24h after collection (43% and 65% on sampling day, 41 and 34% the following day, for cord and maternal samples, respectively). Few samples were received later, and this delay was tracked. The maximal delay for cord blood was 3 days (3 samples) and 5 days (1 sample), and for maternal blood 2 days (3 samples) and 3 days (1 sample).

### 2.3 Laboratory methods

We assessed innate and adaptive immunity of pregnant women at mid-gestation and of their newborn children in plasma, and after direct *ex vivo* activation of whole blood with R848 (Resiquimod, a TLR7/8 ligand) and PHA (phytohaemagglutinin, a selective T cell mitogen), by measuring a large panel of cytokines secreted by monocytes, dendritic cells, granulocytes and T cells.

200 µL of blood was used for cell culture (activation with R848 or PHA), and the other part of blood was centrifuged (800g, 10min), to obtain plasma that was frozen (-80°C). Whole blood cells were activated by incubating 100µL of whole blood with 100µL of culture medium (RPMI supplemented with 10% fetal calf serum, gentamycin, sodium pyruvate and non-essential amino acids) containing R848 (*In vivo*gen, 5µg/mL), or PHA (Oxoid, 10µg/mL). After a 24-hour incubation at 37°C, tubes were centrifuged, and culture supernatants were collected and frozen (-80°C). Culture supernatants or plasma were thawed and their CK contents were measured by cytometric beads arrays (BD™ CBA Human cytokines Flex Set that is a bead-based immunoassay capable of simultaneously measuring several cytokines in biological fluids, BD Biosciences).The following CBA kits were used: RRID: AB_2869127, AB_2869336, AB_2869131, AB_2869130, AB_2869133, AB_2869132, AB_2869177, AB_2869337, AB_2869315, AB_2869137, AB_2869139, AB_2869134, AB_2869161, AB_2869125, AB_2869154, AB_2869128, AB_2869135, AB_2869129), after appropriate dilution of samples (from 1/3 to 1/80), according to manufacturer’s instructions. Acquisition of samples was performed on a BD FACS Canto II flow cytometer, using BD Facs DIVA software. Results were analyzed using BD FCAP array, a software that enables complete analysis of data, and interpolation of sample concentrations by comparison to a standard curve. All recorded values were within the standard curve range. CK dosage was done in separate experiments for mother blood (MB) and cord blood (CB) samples, to lower experimental bias. Maximal and minimal limits of detection were defined in each experiment. IL-5, IL-6, IL-8, IL-9, IL-10, IL-12p70, IL-13, IFNα, IFNγ, MCP1, RANTES, and TNFα were measured at baseline; IL-2, IL-4, IL-9, IL-10, IL-13, IL-17A, IFNα, IFNγ, TNFα were measured after activation with PHA, and IL-1β, IL-6, IL-8, IL-10, IL-12p70 (or p40 for CB), IFNα, IFNγ, and TNFα were measured after activation with R848. After PHA activation, we observed that IFNα was mostly undetectable, and that IL-4 and IL-13 were highly correlated (**Supplementary Figure 2A**), so we gave up measuring IFNα and IL-4. Complete blood counts were also determined automated hematology analyzer (XE-Sysmex), on a separate EDTA blood sample.

### 2.4 Cytokine concentrations coding and standardization

For cytokines with more than 70% of values higher than the limit of detection, concentrations below the limit of detection (LOD) were imputed by values randomly selected between 0 and LOD based on their estimated underlying distribution (26, 27) and concentrations were log10 transformed. Cytokines with more than 30% of values below detection levels were coded as binary variable (not detected vs. detected) and were not considered in the mother-cord blood CK correlations.

Technical variability during the experimentation, either related to the sample analyzed or to the handling conditions in the laboratory, can lead to non-systematic measurement error in the CK concentrations. To limit the effect of this between-sample technical variability, for each of the four analytical set (MB baseline, CB baseline, MB activated, CB activated) we standardized the CK concentrations using a two-step approach when needed (28). Technical factors considered at baseline for CB and MB samples were analytical batch, time from sample collection to sample reception and time from sample receipt to sample determination. Upon activation, technical factors were: experimental batch effect, time from sample collection to sample reception, duration of the activation, R848 or PHA age at the time of sample activation; storage duration and observed quality of the sample (only for CB). These technical variables are described in **Supplementary Table 5**. In a first step, we identified technical factors associated (p < 0.20) to log10 transformed CK concentrations using adjusted linear regression models, and for each CK concentration we estimated the effect of each technical factor identified using a multiple linear regression model (see **Supplementary Tables 6–11**). In the second step, using the measured CK concentration and the estimated effects of each technical factors associated with CK concentrations (p < 0.20), we predicted the corrected CK concentration representing the concentrations that would have been measured without between-sample technical variability.

### 2.5 Statistics

Statistical analyses were performed using the SAS 9.4 statistical software (SAS institute, Cary, NC), R (version 4.1.2) or GraphPad Prism (version 9.1.2) softwares.

Descriptive results are given as means ± SD or as percentages. CK concentrations are described both with percentiles of the raw values and percentiles of the imputed-corrected-log10 transformed values for CK with detection level >70% and in percentage of detection for CK with detection level <70%. All analyses were performed on CK imputed-corrected-log10 transformed values, except in **Supplementary Tables 1**, **2** where raw CK values are described.

#### 2.5.1 Principal components analyses

As an exploratory analysis, for each analytical set, principal components analyses were performed on scaled values of CK concentrations to summarize the main data structure within the data (package prcomp).

#### 2.5.2 Association and correlation analyses

For each analytical set, the association between CK concentrations and leukocyte counts, blood sampling seasons, mother characteristics (age at conception, BMI before pregnancy, smoking during pregnancy, educational level) and child characteristics (sex and gestational age at birth) was estimated, using Pearson correlation, t-test or ANOVA test for continuous, binary and categorical determinants, respectively.

Differences between mothers and children blood cells counts, or CK produced were tested for significance by unpaired or paired student t test, to take into account all values, or only the values for mother-child pairs, respectively. Pearson’s correlation was used for examining parametric correlations between continuous variables. Correlations were represented using the corplot or psych packages. To simultaneously account for all CK secretions in mother-child pairs, the distance between mother and child was calculated as pairwise Mahalanobis distance (able to account for inter-cytokine correlations) using values of all cytokines relevant to the condition (cf above) and common between mother and child (baseline: IL-8, MCP1, RANTES, PHA: IL-2, IL-10, IL-13, IFNγ, R848: IL-1-β, IL-6, IL-8, IL-10, IFNα, TNFα)(performed in base R, using the dist and mahalanobis functions). The Mahalanobis distance is equivalent to Euclidean distance on PCA-rotated, scaled values, and was chosen to account for inter-cytokine correlations. The mean mother-child distance for the actual sample was compared to the mean distance distribution in 10,000 permutations of mother-child pairs. In order to control for cellularity in mother-child CK correlations and distance, the analyses were also performed on CK level adjusted for leucocyte counts (by analyzing the residuals of simple linear regression model between the cytokine levels and leukocyte counts).

#### 2.5.3 Multivariate analyses

The maternal-cord blood CK concentration associations were further studied in multivariate linear regression models (with cord blood CK as outcomes, maternal blood CK as predictors) adjusted on potential confounders identified from the Directed Acyclic Graph (**Supplementary Figure 3**) drawn to determine the variable set to take into account to explain our variables of interest (maternal and child CK). The main models (M1) were adjusted on maternal leukocyte counts (continuous), neonatal blood sampling season (spring, summer, autumn, winter), smoking during pregnancy and child sex. The main models were adjusted on the maternal and not neonatal leukocyte counts, because neonatal leukocyte counts were missing for 32% of the mother-child pairs and these two variables are correlated. Nevertheless in a sensitivity analysis (models M2), models were adjusted on neonatal and not maternal leukocyte counts to assess the robustness of the results.

## 3 Results

### 3.1 Population description

All infants had a birth weight (3301.8 ± 447.2 g) and gestational age at birth (39.7 ± 1.5 weeks of gestation) within normal range (**Table 1**). The average maternal age at delivery was 32.5 ± 3.8 years, and the average pre-pregnancy maternal BMI was 22.5 ± 3.9 kg/m2. Only few mothers smoked during pregnancy (n=12, 4.1%). Fifty-two (16%) of the mothers reported to have ever had asthma. All mothers had an elevated educational level (at least 2 years university level).

**Table 1:** Description of the study population.

	Mother with ≥1 maternal cytokine level, n=322	Infant with ≥ 1 cord blood cytokine level, n=156	Mother- infant pairs with ≥ 1 maternal and cord blood cytokine levels, n=145
Mother age (years) *	32.5 ± 3.8 (322)	32.2 ± 3.8 (156)	32.2 ± 3.8 (145)
Mother body mass index before pregnancy *	22.5 ± 3.9 (319)	22.3 ± 3.3 (154)	22.3 ± 3.4 (143)
Mother active smoking before pregnancy †	6.2 (18)	7.7 (11)	6.8 (9)
Mother active smoking during pregnancy †	4.1 (12)	7.4 (11)	7.2 (10)
Maternal educational level:			
2 years after high schoo l†	15.0 (48)	16.2 (25)	15.4 (22)
3 to 4 years after high school †	25.3 (81)	26.6 (41)	25.9 (37)
5 years or more after high school†	59.7 (191)	57.1 (88)	58.7 (84)
Maternal asthma †	16.1 (52)	15.4 (24)	15.9 (23)
Maternal rhinitis †	41.0 (132)	37.8 (59)	37.9 (55)
Gestational age at maternal plasma sample (weeks) *	20.3 ± 3.9 (278)	20.1 ± 3.7 (126)	20.1 ± 3.7 (126)
Gestational age at birth (weeks) *	39.7 ± 1.5 (321)	39.9 ± 1.1 (156)	39.9 ± 1.1 (145)
Leucocyte in maternal sample (G/L) *	9.0 ± 2.0 (311)	--	9.0 ± 2.0 (141)
Leucocyte in cord-blood sample (G/L) *	--	14.4 ±4.3 (105)	14.4 ±4.3 (99)
Season of maternal plasma sample:			
Autumn †	19.3 (62)	--	22.1 (32)
Spring †	30.4 (98)		34.5 (50)
Summer †	27.9 (90)		24.8 (36)
winter †	22.4 (72)		18.6 (27)
Season of cord-blood sample:			
Autumn †	--	33.3 (52)	34.5 (50)
Spring †		21.8 (34)	20.7 (30)
Summer †		28.2 (44)	29.0 (42)
winter †		16.7 (26)	15.9 (23)
Delivery by C-section †	13.7 (44)	10.3 (16)	9.7 (14)
Birth weight (g) *	3302 ± 447 (321)	3282 ±427 (156)	3285 ± 422 (145)
Child sex (males) †	53.0 (170)	48.1 (75)	46.9 (68)

*: mean±sd (n).

† : % (n).

### 3.2 Higher innate and lower adaptive cytokines levels in cord blood compared to maternal samples

**Figure 1:**
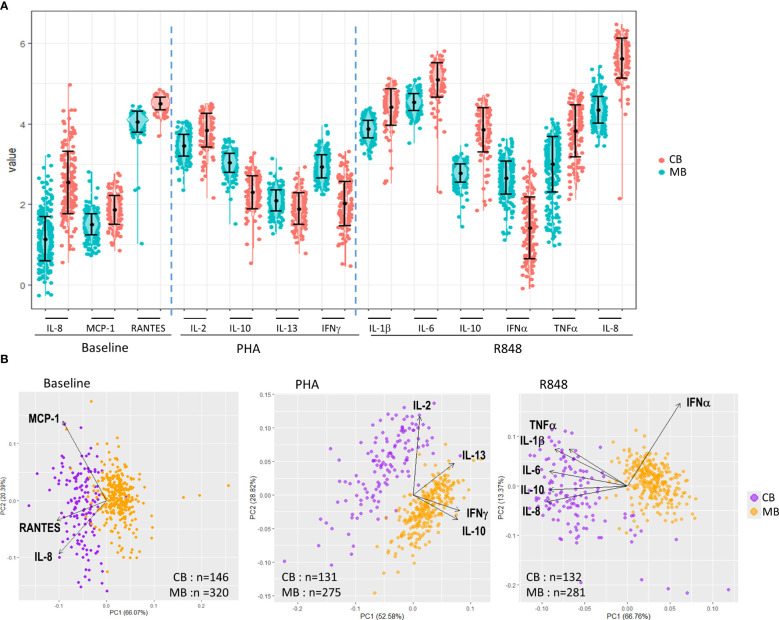
Description of cytokine and chemokine production in whole blood from pregnant women and neonates at baseline and upon activation. **(A)** scatter plots showing mean and standard deviation of cytokines and chemokines (log10 transformed) for all mothers (blue dots, MB) and children (red dots, CB) measured at baseline (left) and after activation of whole blood by PHA (middle) or R848 (right). CK levels were significantly higher in samples from neonates compared to mothers, except for IL-10, IL-13 and IFNγ after PHA and IFNα after R848 (p < 0.001, student t test). **(B)** Principal component (PC) analysis of the immune response for all mothers (yellow dots) and children (purple dots) measured at baseline (left) and after activation of whole blood by PHA (middle) or R848 (right). Each dot represents one subject, and analyses were restricted to CK with detection level >70%. The loadings represented by arrows show how (the direction) and how much (the length) each CK contributes to PC1 and PC2.

CK levels were very low in plasma at baseline, with concentrations above the limit of detection (LOD) for more than 70% of the samples for 4/11 cytokines in cord blood (IL-8, IL-12p40, MCP1 and RANTES), and 3/12 in maternal blood (IL-8, MCP1 and RANTES) (**Supplementary Tables 1**, **2**). As shown in **Figure 1A**, at baseline, statistically higher concentrations of IL-8, MCP1 and RANTES were found in CB compared to MB.

Activation of whole blood cells by PHA or R848 resulted in large CK secretion, with specific statistically significant differences between cord and maternal blood samples. Following activation by PHA, CB contained higher levels of IL-2 than MB, but lower levels of IL-10, IL-13 and IFNγ (**Figure 1A**). Activation by R848 led to higher concentrations of IL-1β, IL-6, IL-10, TNFα and IL-8 in CB compared to MB, and only IFNα was found at lower concentration in CB compared to MB.

Principal component analyses performed in each experimental condition show that MB and CB samples separated in two highly distinct clusters (**Figure 1B**). At baseline, CB contained higher levels of RANTES, MCP1 and IL-8 compared to MB. After activation with PHA, CB differed from MB through their higher secretion of IL-2, but lower secretion of IFNγ and IL-10. The activation by R848 led to lower secretion of IFNα in CB, but higher production of IL-1β, IL-6, IL-8, IL-10 and TNFα compared to MB.

Overall these analyses indicate that cord blood and maternal samples display specific profiles in the innate and adaptive compartments at baseline and upon activation.

### 3.3 CK levels within maternal and cord blood samples at baseline and after activation are highly correlated

**Figure 2:**
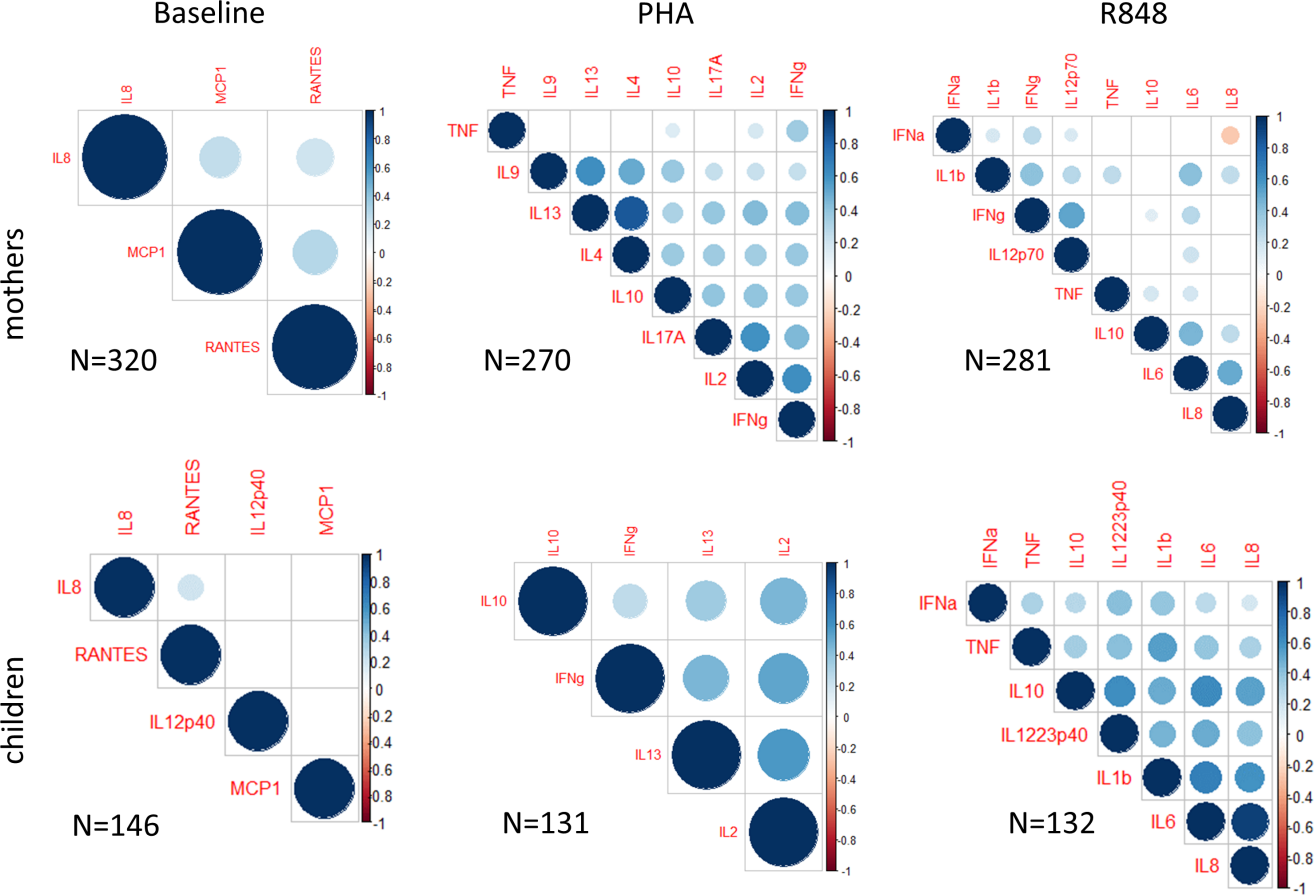
Cytokine and chemokine covariation. Pairwise Pearson’s correlations between CK with hierarchical clustering. Plots for all mothers (higher panels) and children (lower panels) CK measured at baseline (left) and after activation of whole blood by PHA (middle) or R848 (right). Pearson’s r values are indicated with size and color scale only when p values were below 0.05. Numbers of analyzed mother-children pairs are shown on each panel.

We then analyzed correlations between cytokines in maternal samples, and describe here the significant correlations observed (p<0.05) (**Figure 2**, upper panels). At baseline, moderate correlations were observed between IL-8, RANTES and MCP1 levels in MB (0.2<r<0.3). After PHA activation, high correlations (r>0.5) were observed between IL-9 and IL-13 while IL-2 and IFNγ correlated with each other. Following R848 activation, the highest correlations (0.4<r ≤ 0.5) were observed for IL-6 that was correlated with IL-10, IL-8 and IL-1β, while IFNγ was correlated with IL-12p70. Of note, all these correlations but one (IL-8/IFNα, r= - 0.27, **Supplementary Figure 2B**) were positive.

In CB (**Figure 2**, lower panels), only a slight correlation was observed between IL-8 and RANTES at baseline. Interestingly, in activated CB samples, all CK were correlated, and several CK showed high correlations (0.5<r<1) with each other. For example, after activation with R848, IL-6, IL-8 and IL-1β were highly correlated with one another (r≥0.6), and IL-10 was correlated with IL-12/23p40, IL-6 and IL-8 (r>0.5). After activation with PHA, IL-2 production was highly correlated (r>0.5) with IFNγ and IL-13.

### 3.4 CK levels in maternal and cord blood are associated with leukocyte counts, maternal body mass index, age, smoking and sampling season

We then examined individual and external factors that could impact CK levels in maternal and cord blood samples. Cell counts were significantly higher in MB compared to CB (9.07 ± 2.1 vs. 14.10 ± 4.6 million WBC per mL, respectively, p<0.0001 considering all samples (respectively n=219 and n=436), or paired samples (n=205) with unpaired or paired t test, respectively), **Supplementary Figure 4A**).

Leukocyte counts were highly correlated with CK levels in activated maternal (PHA: IL-2, IL-9 and IL-13; R848: IL-1β, IL-6, IL-8, IL-10, IFNα and IFNγ) or cord blood (R848: IL-6, IL-8 and IL-10) samples (**Table 2**). Mothers with high BMI (before pregnancy) had higher CK content at baseline (RANTES), and also after activation with PHA (IL-2 and IL-13), and R848 (IL-6 and IL-10) (**Table 2**). CK secreted in CB samples were not correlated with pre-pregnancy maternal BMI, nor with birth weight or maternal weight measured during the 3^rd^ trimester (not shown). In activated samples, IL-17A (PHA) and IFNα (R848) were lower in samples obtained from older pregnant women. Gestational age at birth was not correlated with cord blood cytokines. In maternal samples, RANTES (NA) and IL-6 (R848) were correlated with gestational age at sampling date (**Supplementary Table 4**).

**Table 2:** Correlations between cytokine and chemokine secretions and leukocyte counts, maternal BMI and maternal age.

	Leukocyte counts		Maternal BMI †		Maternal age †	
	Maternal	Cord blood	Maternal	Cord blood	Maternal	Cord blood
Basal						
n	309	97-105	317	144-154	320	146-156
IL8	0.11.	0.08	-0.08.	-0.03	0.05	-0.05
IL12§		-0.09		0.06		0.02
MCP1	0.05	-0.33***	0.08	0.06	0.02	0.03
RANTES	0.02	0,00	0.12*	0.02	-0.08	-0.03
PHA						
n	216-270	84	217-272	128	220-275	131
IL2	0.24***	0.15.	0.12*	0,00	-0.02	-0.11
IL4	0.03		0.02		0.06	
IL9	0.23***		0.1.		-0.03	
IL10	0.04	-0.06	0.02	-0.11	-0.08	-0.02
IL13	0.25***	0.22.	0.18**	-0.11	0.01	-0.02
IL17A	0.07		0.05		-0.12*	
IFNg	0.13.	-0.13	0.08	-0.14	-0.07	-0.01
TNF	0.06		0.03		-0.05	
R848						
n	275	85	278	129-131	281	132
IL1b	0.38***	0.20.	0.07	-0.08	-0.01	0.01
IL6	0.36***	0.33***	0.12*	-0.04	0.01	0.01
IL8	0.36***	0.26***	0.09	-0.05	0.05	0.01
IL10	0.30***	0.43***	0.13*	-0.01	-0.1.	0.04
IL12§	0.11.	0.02	-0.02	-0.02	0.04	0.04
IFNa	0.13*	0.02	0.11.	0,00	-0.18**	-0.05
IFNg	0.24***		0.07		-0.05	
TNF	0.03	0.1	0.09	-0.05	0.1.	-0.01

Pearson r correlations between continuous variables and cytokines levels, Bold: p <0,05.

p value: . <0,1; *≤0,05; **≤0,01; ***≤0,001.

§: IL12p70 for maternal samples and IL12p40 for cord blood samples.

†: BMI before pregnancy, age at conception.

Some CK levels were associated with sampling season (**Supplementary Table 3**). In MB, IL-8 content was higher at baseline in summer, while IL-9, IL-13 and IL-17A were higher in PHA-activated autumnal samples, and IFNα (R848) was higher during winter. Tobacco smoking (**Tables 3**, **4**) was associated with higher IL-9 (PHA), IL-13 (PHA) and IL-10 (R848) secretion in MB, as well with higher IFNα and TNFα secretion upon R848 activation in CB. Child sex did not modify maternal cytokine profile, but we observed a trend towards lower IL-10 production upon PHA activation in CB from male children (p=0.07).

**Table 3:** Difference between cytokine and chemokine secretions according to maternal smoking history .

	Smoking during pregnancy							
	Maternal				Cord blood			
	n	NO	YES	p	n	NO	YES	p
Basal								
IL8	292	1.13 ± 0.55	1.14 ± 0.4	0.98	148	2.56 ± 0.8	2.33 ± 0.64	0.35
IL12§					148	2.07 ± 0.23	1.92 ± 0.17	**0.05**
MCP1	292	1.5 ± 0.26	1.59 ± 0.13	0.24	138	1.86 ± 0.35	1.74 ± 0.29	0.26
RANTES	292	4.04 ± 0.27	4.14 ± 0.16	0.22	148	4.51 ± 0.14	4.41 ± 0.27	**0.04**
PHA								
IL2	252	3.46 ± 0.27	3.6 ± 0.25	0.1	128	3.82 ± 0.43	4.04 ± 0.27	0.14
IL4	199	1.96 ± 0.26	2.14 ± 0.35	*0.07*				
IL9	252	1.63 ± 0.32	1.87 ± 0.32	**0.02**				
IL10	252	3.02 ± 0.25	3.16 ± 0.24	*0.07*	128	2.28 ± 0.42	2.36 ± 0.33	0.55
IL13	252	2.08 ± 0.26	2.36 ± 0.31	**0.001**	128	1.87 ± 0.39	1.9 ± 0.37	0.87
IL17A	247	2.56 ± 0.34	2.68 ± 0.31	0.28				
IFNg	252	2.93 ± 0.29	3.09 ± 0.28	*0.09*	128	1.99 ± 0.55	2.23 ± 0.53	0.21
TNF	252	2.51 ± 0.73	2.12 ± 1.01	0.11				
R848								
IL1b	258	3.88 ± 0.22	3.87 ± 0.21	0.83	130	4.41 ± 0.43	4.67 ± 0.37	*0.07*
IL6	258	4.53 ± 0.22	4.57 ± 0.2	0.55	130	5.08 ± 0.44	5.23 ± 0.29	0.33
IL8	258	4.36 ± 0.33	4.41 ± 0.31	0.58	130	5.63 ± 0.5	5.66 ± 0.35	0.82
IL10	258	2.76 ± 0.23	2.97 ± 0.27	**0.005**	128	3.84 ± 0.55	4.08 ± 0.33	0.21
IL12§	258	1.35 ± 0.37	1.32 ± 0.39	0.84	128	3.48 ± 0.45	3.66 ± 0.26	0.24
IFNa	258	2.67 ± 0.42	2.67 ± 0.26	0.96	128	1.38 ± 0.74	1.95 ± 0.91	**0.03**
IFNg	258	2.55 ± 0.5	2.54 ± 0.39	0.95				
TNF	258	3 ± 0.7	2.81 ± 0.77	0.39	130	3.8 ± 0.63	4.27 ± 0.64	**0.03**

mean±SD of cytokine content per group.

§: IL12p70 for maternal samples and IL12p40 for cord blood samples.

bold: p value ≤0.05; italic: 0.05 <p ≤0.1.

**Table 4:** Difference between cytokine and chemokine secretions according to child gender .

	Child sex							
	Maternal				Cord blood			
	n	Female	Male	p	n	Female	Male	p
Basal								
IL8	319	1.16 ± 0.56	1.12 ± 0.53	0.14	156	2.5 ± 0.77	2.58 ± 0.79	0.54
IL12§					156	2.02 ± 0.23	2.08 ± 0.22	*0.1*
MCP1	319	1.49 ± 0.27	1.51 ± 0.25	0.43	146	1.81 ± 0.35	1.91 ± 0.35	*0.08*
RANTES	319	4.04 ± 0.2	4.04 ± 0.31	0.42	156	4.51 ± 0.15	4.5 ± 0.17	0.47
PHA								
IL2	275	3.44 ± 0.27	3.48 ± 0.27	0.23	131	3.85 ± 0.39	3.82 ± 0.46	0.63
IL4	220	1.94 ± 0.31	1.97 ± 0.23	0.39				
IL9	275	1.64 ± 0.34	1.64 ± 0.3	0.93				
IL10	281	3.01 ± 0.24	3.04 ± 0.25	0.36	131	2.37 ± 0.33	2.21 ± 0.47	**0.02**
IL13	275	2.09 ± 0.29	2.09 ± 0.24	0.98	131	1.92 ± 0.36	1.84 ± 0.43	0.26
IL17A	270	2.54 ± 0.32	2.56 ± 0.35	0.54				
IFNg	275	2.91 ± 0.29	2.96 ± 0.29	0.22	131	2 ± 0.55	2.02 ± 0.57	0.84
TNF	275	2.48 ± 0.7	2.5 ± 0.76	0.81				
R848								
IL1b	281	3.85 ± 0.24	3.89 ± 0.2	0.11	134	4.46 ± 0.46	4.38 ± 0.45	0.3
IL6	281	4.52 ± 0.23	4.54 ± 0.21	0.54	134	5.09 ± 0.47	5.08 ± 0.39	0.98
IL8	281	4.35 ± 0.35	4.35 ± 0.32	0.99	134	5.64 ± 0.54	5.61 ± 0.44	0.71
IL10	281	2.77 ± 0.24	2.77 ± 0.23	0.93	132	3.9 ± 0.42	3.79 ± 0.65	0.25
IL12§	281	1.36 ± 0.36	1.31 ± 0.38	0.22	132	3.47 ± 0.37	3.48 ± 0.51	0.93
IFNa	281	2.64 ± 0.39	2.67 ± 0.43	0.54	132	1.29 ± 0.75	1.53 ± 0.76	*0.07*
IFNg	281	2.51 ± 0.48	2.58 ± 0.49	0.20				
TNF	281	2.91 ± 0.76	3.06 ± 0.63	*0.08*	134	3.85 ± 0.64	3.78 ± 0.65	0.58

mean±SD of cytokine content per group.

§: IL12p70 for maternal samples and IL12p40 for cord blood samples.

bold: p value ≤0.05; italic: 0.05 <p ≤0.1.

As cellularity was different between maternal and cord blood, and was highly correlated to CK levels, we evaluated whether the differences observed between maternal and cord blood CK levels remained similar, taking cellularity into account. Differences between MB and CB CK concentrations remained when considering CK levels pre-adjusted for leukocyte counts (**Supplementary Figure 4B**), suggesting that the overall increased basal and R848 inflammatory response observed in CB samples is not only due to their higher cell counts but also reflects a higher cytokine production per cell. For IFNα (R848), and IL-13, IL-10 and INFγ (PHA), the higher response of MB compared to CB was maintained. Similarly, most of the previous described correlations between CK in maternal or cord blood samples remained significant, taking cellularity into account (**Supplementary Figure 5**).

### 3.5 Cord blood and maternal CK productions at baseline or upon activation are correlated

**Figure 3:**
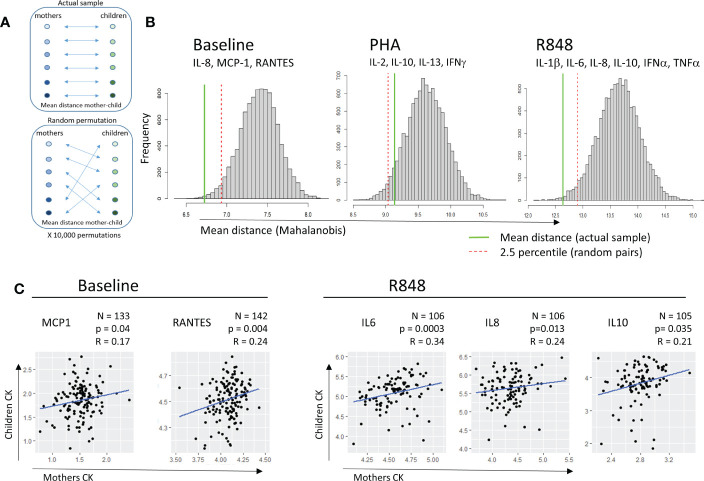
Correlation between mother and child cytokine levels. **(A)** Multivariate distance between mother and child: the mean pairwise Mahalanobis distance between each mother and her child was calculated, using cytokine levels of relevant cytokines for each condition (subtitled in B). The mean distance was compared to the mean distance distribution in 10,000 random permutations of mother-child pairs. **(B)** Histograms of mean distances in random permutations. The red dotted line indicates the 2.5% percentile of mean distances in the permutations, while the green line indicates the mean distance mother-child in the actual sample. **(C)** Correlation between levels of cytokines for mother and child at baseline (left, MCP1, RANTES) or upon R848 stimulation (right, IL-6, IL-8, IL-10). Pearson’s correlation coefficients and p-values are displayed for each cytokine.

To analyze associations between maternal and cord blood immune responses, we calculated the mean distance between paired maternal and CB in multivariate CK space, at baseline and after activation, and compared this distance with 10,000X randomized pairs of mothers and children (**Figure 3A**).

These distances were calculated using the pairwise Mahalanobis method (**Figure 3B**) to account for correlated variables. At baseline and after R848 activation, but not after PHA activation, the mean distance between mothers and their children (green solid line) was shorter than the mean distance between randomized mother-child pairs (distance in the 2.5 shortest percentile, red dotted line), suggesting a global association between maternal and neonate immunity. The pairwise Mahalanobis distance still showed an association between maternal and cord blood CK at baseline and after R848 activation after adjustment for cellularity (**Supplementary Figure 6**).

Regarding each CK separately, no correlations between MB and CB matched samples were found for CK secreted after PHA activation (data not shown). At baseline, moderate positive correlations between MB and CB samples were observed for MCP1 (r=0.19, p=0.02) and for RANTES (r=0.21, p=0.01). After activation by R848, positive correlations were observed for IL-6 (r=0.34, p<0.001), IL-8 (r=0.24, p=0.01) and IL-10 (r=0.21, p=0.03) between MB and CB (**Figure 3C**).

### 3.6 Maternal CK levels are independent predictors of neonate CK levels

For CK showing maternal-neonatal correlation, we further investigated the maternal and child CK association using multivariable regression model to account for potential confounding factors (MB leucocyte counts, maternal BMI, maternal smoking, birth season, and child sex). In this analysis performed on 91 to 132 mother-child pairs, neonates’ baseline MCP1 and RANTES levels, and R848 IL-6, IL-8 and IL-10 levels remained associated with maternal corresponding CK (**Table 5**). In a M2 model, adjusted on CB leucocyte counts, maternal BMI, maternal smoking, birth season, and child sex (n=60 to 95 mother-child pairs), neonates’ baseline RANTES level, and R848 IL-6, and IL-8 levels remained associated with maternal CK (data not shown).

**Table 5:** Associations between maternal and cord blood CK secretions adjusted for cellularity, season and body mass index.

	Baseline						R848								
	Cord blood MCP-1 (n=122)			Cord blood RANTES (n=132)			Cord blood IL10 (n=91)			Cord blood IL6 (n=93)			Cord blood IL8 (n=93)		
	Beta	95%CI	p	Beta	95%CI	p	Beta	95%CI	p	Beta	95%CI	p	Beta	95%CI	p
Maternal CK *	0.34	0.10 ; 0.57	**0.01**	0.16	0.04 ; 0.28	**0.01**	0.58	0.10 ; 1.05	**0.02**	0.62	0.14 ; 1.09	**0.01**	0.41	0.06 ; 0.76	**0.02**
Maternal leucocytes counts	0,00	-0.03 ; 0.03	0.9	-0.01	-0.02 ; 0.00	*0.06*	-0.04	-0.10 ; 0.02	0.21	-0.01	-0.07 ; 0.05	0.75	0,00	-0.07 ; 0.07	0.98
Birth season: Season, autumn	-0.16	-0.34 ; 0.03	0.19	0.04	-0.03 ; 0.12	**0.05** ; 0.23	0.08	-0.26 ; 0.43	0.9	0.11	-0.18 ; 0.39	0.57	-0.02	-0.36 ; 0.31	0.57
Season, spring	-0.13	-0.34 ; 0.07		0.02	-0.06 ; 0.10	0.59	0.12	-0.26 ; 0.49		0.03	-0.29 ; 0.35		-0.13	-0.5 ; 0.24	
Season, Summer	-0.02	-0.21 ; 0.16		0.09	0.02 ; 0.17	**0.01**	0.12	-0.21 ; 0.46		-0.06	-0.34 ; 0.23		-0.18	-0.51 ; 0.15	
Smoking YES	-0.15	-0.38 ; 0.08	0.19	-0.05	-0.15 ; 0.04	0.25	0.04	-0.37 ; 0.45	0.85	0.07	-0.28 ; 0.42	0.69	0.07	-0.34 ; 0.49	0.72
Child sex F	-0.11	-0.22 ; 0.01	0.08	0.01	-0.04 ; 0.05	0.75	0.12	-0.10 ; 0.33	0.28	0.01	-0.17 ; 0.19	0.89	0.03	-0.19 ; 0.24	0.81

* Cytokine or chemokine in maternal variable is the same as evaluated as X variable in cord blood sample.

The p value of the F test (type III Sum of squares) for inclusion of the factor birth season is indicated in a separate line. If significant, the p-value of the t-test for each season coefficient is indicated below. Reference: winter.

bold: p value ≤0.05.

## 4 Discussion

To our knowledge, this is the first study to address the maternal-neonate immune function in a large population-based population, by considering a wide range of CK measured at baseline and after activation of innate blood immune cells. For the CK with frequencies of detection above 70% in both MB and CB (n=13), our study shows strong mother-child associations for MCP1 and RANTES measured at baseline in plasma and for IL-6, IL-8 and IL-10 upon R848 stimulation, demonstrating an early and *in utero* imprinting of child immunity.

### 4.1 Description of neonatal and maternal immune responses

Our results showed that activation of T cells in whole CB induced high levels of IL-2, but low levels of IFNγ, IL-13 or IL-10, in comparison to MB samples, despite CB cell counts being higher than MB cell counts. These results converge with previous studies indicating that the neonate adaptive immune system is immature, their T cells are naive, thus although they can secrete high amounts of IL-2, they usually produce few Th1 or Th2 cytokines (29). Upon activation with the TLR7/8 ligand R848, that activates plasmacytoid and myeloid dendritic cells, but also granulocytes and monocytes (30), we observed that neonatal innate immune responses differ from maternal responses, with very low production of IFNα, while productions of IL-1β, IL-6, IL-8, IL-10 and TNFα were very high, in accordance with previous studies (31, 32). These results could be related to the low capacity of neonatal PDC to secrete IFNα (33), due to impaired IRF-7 translocation (34). The higher production of inflammatory CK observed are only partially due to higher cellularity in CB compared to MB. In the literature, increased innate responses in CB were also observed in response to LPS with large secretions of IL-6, IL-8, and IL-10 (35–37), but contradictory results were found when experiments were performed on PBMC, or depending on the method used to measure cytokine production (16). Altogether, this suggests that neonates may rely on a strong inflammatory response to react against infections, and show that this increased inflammatory response relies in part on the higher cell numbers that are present in CB compared to adult blood.

The strong correlations observed between most CK after activation with PHA, which is in agreement with a previous study in pregnant women (38), indicate that some samples produced high levels of all cytokines, while others produce low levels. These correlations were maintained when controlling for cell counts, which means that there was coordinated regulated expression of these cytokines by all T-cells and other effector cells such as NK cells.

Through TLR8 triggering, R848 drives strong production of NF-κB–dependent inflammatory cytokines (30), particularly from myeloid cells. R848 also activates PDC through TLR7 triggering inducing both inflammatory cytokines and IFNα, in relationship to dual IRF7 and NF-kB activation. In whole blood assays, indirect activation may also happen, with secondary responses occurring, such as IFNγ production by NK cells that might be induced by PDC-secreted IFNα. The numerous correlations we observed are in line with previous work describing the activation of common signaling pathways (such as TLR activation of NF-kappa B) co-regulating a typical set of cytokines through transcription factor activation and other mechanisms (e.g. IFN signaling can drive IL-12p35 transcription and formation of IL-12p70) (39, 40), in different cell subsets, thereby ensuring an integrated response against microbial threats (1, 7, 10) While most cytokines were correlated with each other in CB samples, these correlations are less frequent in MB samples, suggesting a higher mechanistic or cellular diversity in cell responses.

Strikingly, we observed an inverse correlation between IL-8 and IFNα secretion following R848 activation in maternal samples. This could rely on TLR7 and TLR8 distinct cell expression patterns, and on their differential downstream signaling pathways, as TLR8 shows a bias toward NF-κB activation while TLR7 shows a dual IRF/NF-κB activation (30). Another explanation may rely in the cross-inhibition of type I interferon intracellular signaling upon cell maturation induced by NF-κB (41).

### 4.2 Factors that modulate maternal and neonates immune responses

We analyzed which factors, besides leucocyte counts, could influence cytokine secretion in maternal or cord blood. Even though correlations coefficients were rather low, we detected significant correlations, and found that mothers with high BMI before pregnancy, or the few mothers who smoked tend to have higher levels of some CK, as previously observed (8, 42). An influence of sampling season was also observed, for maternal and to a lesser extend for cord blood CK. More precisely, our results show increased autumnal secretions for activated maternal samples (IL-9, IL-13, IL-17A (PHA) and IL-1β and IFNα (R848)). Seasonal variation of immune function in healthy adults has been described (43), and may be related to season-related exposure to infections or allergens, but also to physical factors such as daylight duration or temperature. Two studies already described seasonal variations in infant immune responses, but differences in the experimental protocols preclude comparison of detailed variations (44, 45). Gestational age did not influence cord blood cytokines but influenced slightly RANTES (NA) and IL6 (R848) maternal cytokines. Such low impact of gestational age could be related to the low variation for this parameter in our data set compared to other data set focusing on pre-term birth or describing immunity across pregnancy (42, 46).

### 4.3 Correlations between maternal and neonates immune responses

Interestingly, we observed that besides maternal and external factors, CK productions in children at baseline, and after R848 activation, were correlated with maternal CK productions, revealing a strong prenatal immune imprinting influencing immune responses in infants. This imprinting might be due to environmental, hormonal or shared genetic factors, and the relative contribution of these different factors remains to be analyzed. In previous studies focused on atopic mothers, correlations were observed between maternal and children adaptive immune responses determined at the time of delivery (17, 19, 35). We did not find such correlations in our cohort after PHA activation since it contained few mothers with atopy (as compared to the previous studies that were enriched with atopic mothers) that could influence T cell responses in children. In our study, maternal samples were taken during the second trimester of pregnancy, more than 3 months before delivery and CB collection. To our knowledge, there is no data describing such correlations in innate immune system in similar settings. The distance in time between maternal and CB sampling demonstrates that immune imprinting is very strong, and independent of acute maternal exposure to allergen or viruses. This observed phenomenon may have important repercussions, as it means that the neonates’ ability to initiate immune responses is highly influenced by maternal immune capacities, and may be involved in inter-individual differences in response to early infections. Understanding immune system development early in life is important to enhance protection of infants from infections, and develop more efficient vaccines. Recent data suggest that although the child immune system differs at birth, there is a convergence onto a shared trajectory during the first months of life, likely reflecting maturation of the immune system towards an adult state (47) (which does not preclude familial or environmental influences). Several lines of evidence suggest that maternal immune factors (allergic or inflammatory state) can have an impact on child development, leading to an increased risk of developing asthma, allergies, obesity, or neurological disorders (48–50). Understanding inter-individual differences in disease susceptibility remains difficult, and deciphering the relative impact of *in utero* immune imprinting and early life exposure to environmental factors on adult immune capacity is still challenging. The mechanisms responsible for this imprinting are yet to be identified; they could involve genetic-, epigenetic- or environmentally-induced modulations of child innate immune cells. Indeed, epigenome alterations in neonates, modifications in airway microbiota and susceptibility to asthma in children were shown to be associated with maternal prenatal immune status (20). As suggested by Apostol et al., fetal hematopoietic stem cells could be subject to inflammatory conditioning *in utero*, leading to long-lasting changes to immunity, impacting immune functions throughout lifespan (51).

### 4.4 Limits and conclusions

The originality of the SEPAGES cohort relies in the gathering of a homogeneous group of healthy pregnant women, with a rather high education level, and normal BMI. This cohort is one of the first large scale study of innate and adaptive immunity in mothers and neonates. In this regard, the SEPAGES cohort differs from several cohorts or smaller studies developed to assess immune correlates during pregnancy (8, 17, 18, 44, 52, 53), as it comprised few atopic mothers (asthma or allergy), obese or smokers, as compared to previous studies. The size and design of the cohort were instrumental in identifying specific maternal and neonatal immune profiles at baseline and upon immune activation, and in disentangling the role of biological and lifestyle variables that influence these profiles. A limitation of this study is related to the method used for cytokine detection that did not allow quantification of several cytokines at baseline. Accurate measurement would require more sensitive techniques, adapted to the very low cytokine levels in plasma. One of the most striking observations was the correlation between immune function in mothers and their child, the basis of which is as-yet unknown. In the SEPAGES cohort, additional data were collected, including exposure to pollutants and endocrine disruptors, or immune cell phenotypes by multicolor flow cytometry directly ex vivo. These measures will be integrated with the current data to better characterize maternal and neonatal immunity, and to identify environmental factors affecting innate and adaptive immunity during pregnancy and at birth. The availability of frozen cells from all participant in this study will also allow further in-depth analyses to pursue investigations and study the genetic or epigenetic underpinnings of the associations reported here.

## Data availability statement

The original contributions presented in the study are included in the article/**Supplementary Material** further inquiries can be directed to the corresponding author.

## Ethics statement

The studies involving human participants were reviewed and approved by the Commission Nationale de l’Informatique et des Libertés (CNIL, the French data privacy institution, authorization n°2014-263, 2014-06-26), the CPP (Comité de Protection des Personnes Sud-Est V, authorization n°13-CHUG-44, 2014-02-25) and the Agence nationale de sécurité du médicament et des produits de santé (ANSM, authorization n°2013-A01491-44, 2013-12-17). Written informed consent to participate in this study was provided by the participants’ legal guardian/next of kin.

## Author contributions

OM supervised and performed statistical analyses and wrote the manuscript. KU and YM performed and analyzed the experiments. AB prepared the dataset and performed multivariate statistical analyses. SL-C coordinated data and sample collection, SB contributed to data collection. RS conceived the project and obtained funding. CP conceived the project and contributed to data collection. VS conceived the project, supervised statistical analyses, participated to the results interpretation and worked on the manuscript. LC designed and supervised the project and wrote the manuscript. All authors contributed to the article and approved the submitted version.
