# Supplementary figures and images for: Maternal imprinting and determinants of neonates’ immune function in the SEPAGES mother-child cohort
Source: Front Immunol. 2023 Apr 4;14:1136749. doi: 10.3389/fimmu.2023.1136749 (PMC10111372; doi:10.3389/fimmu.2023.1136749)

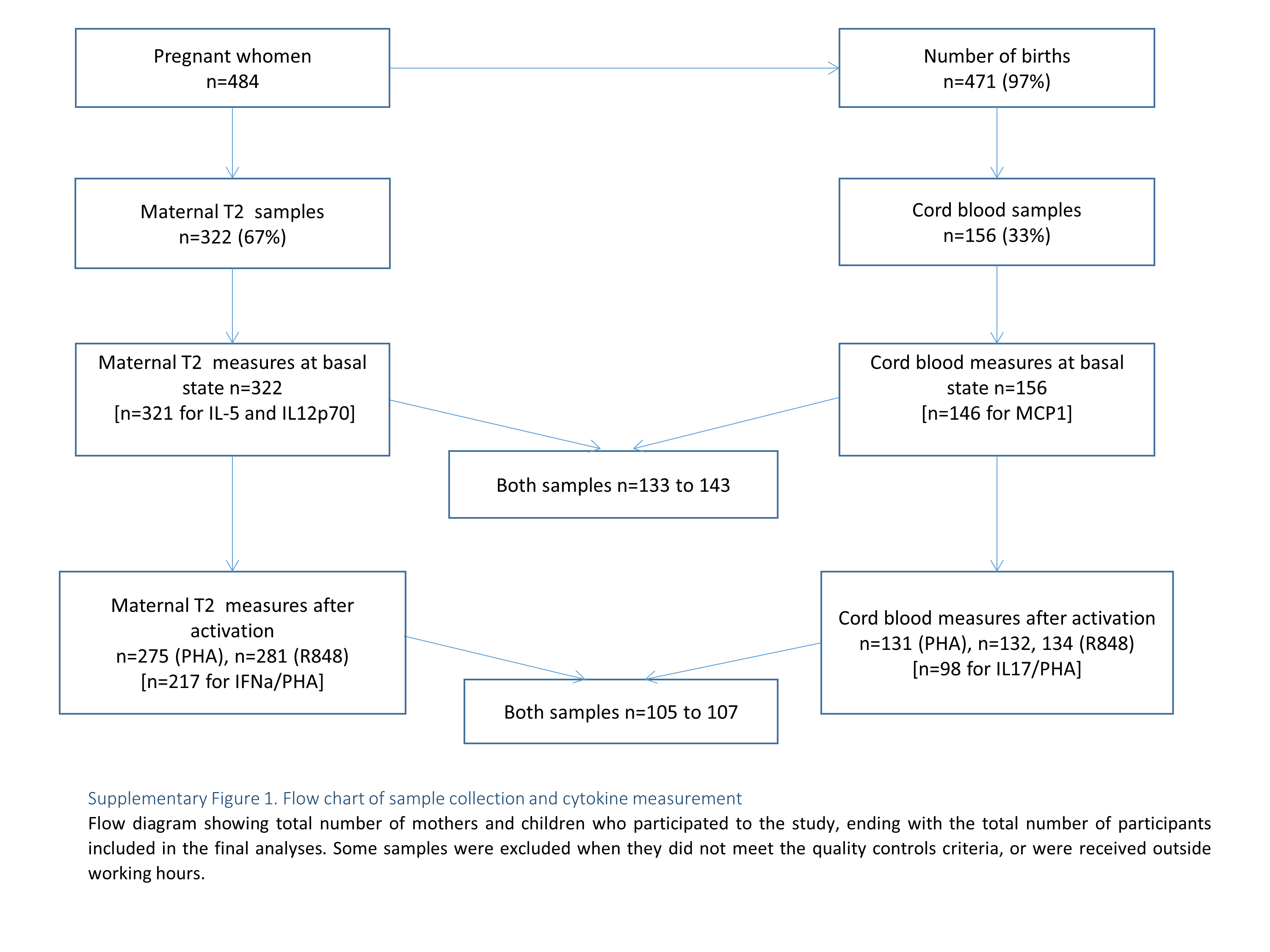

Supplement: Supplementary file 1 [file Image_1.tif]

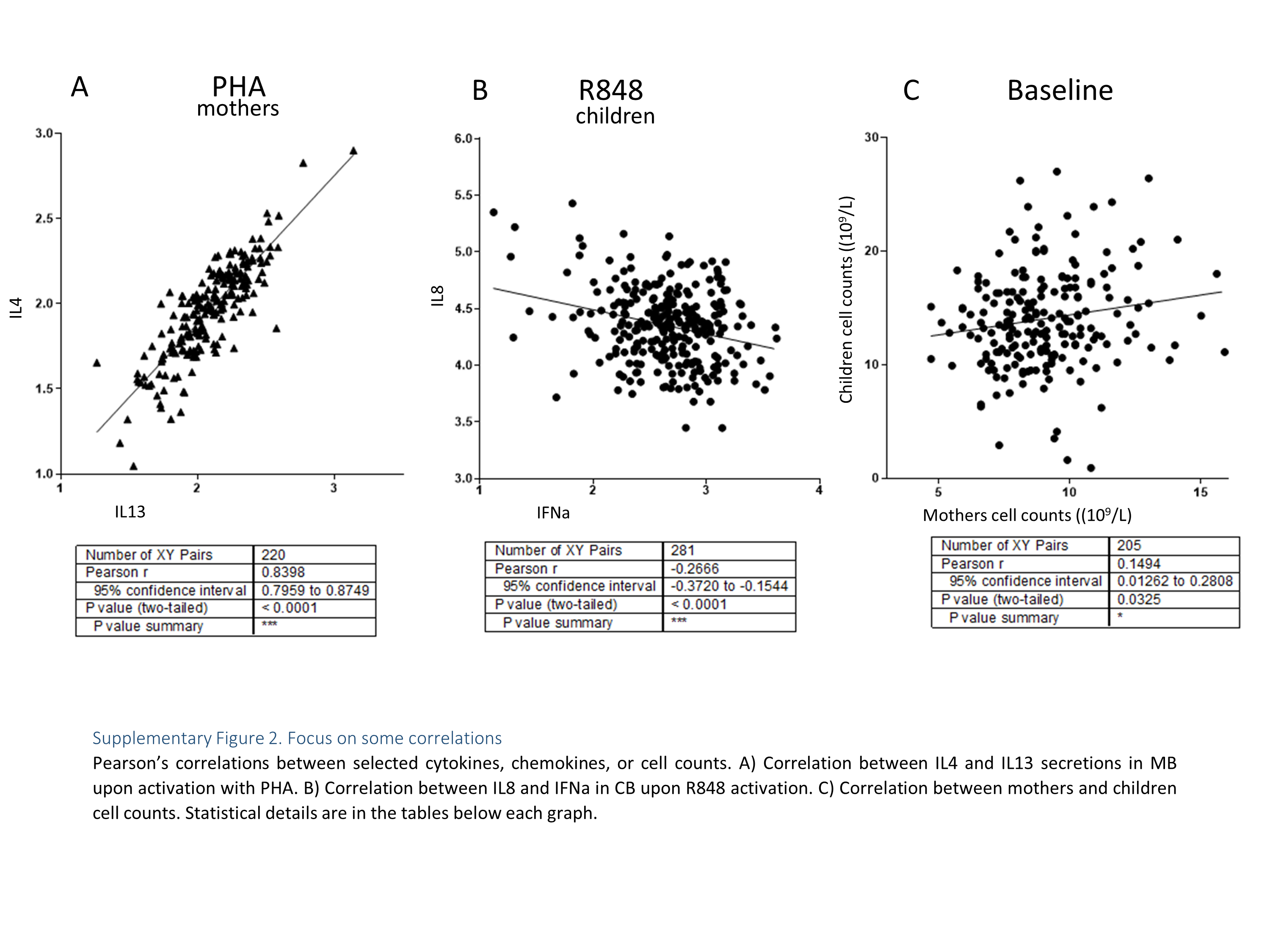

Supplement: Supplementary file 2 [file Image_2.tif]

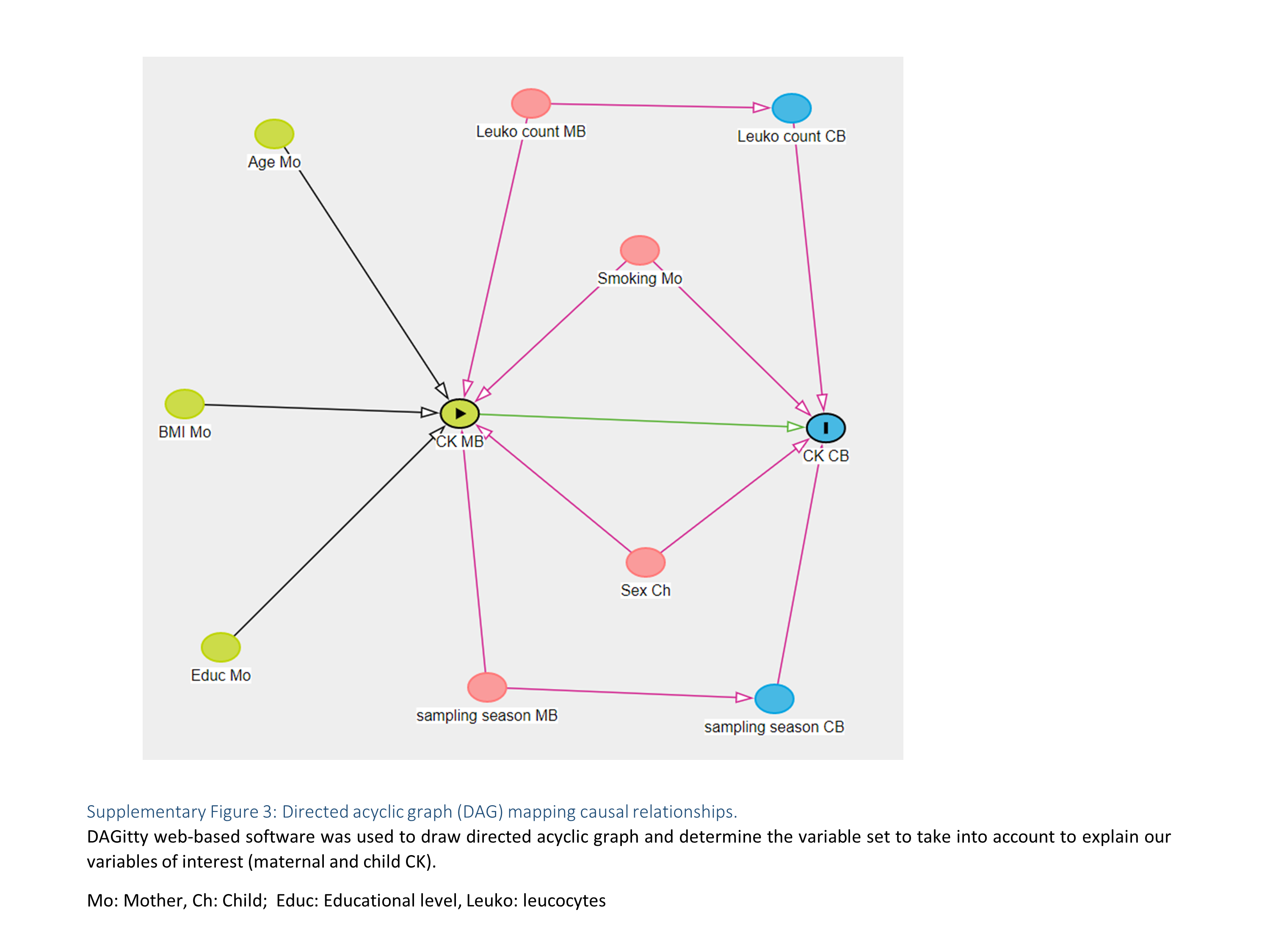

Supplement: Supplementary file 3 [file Image_3.tif]

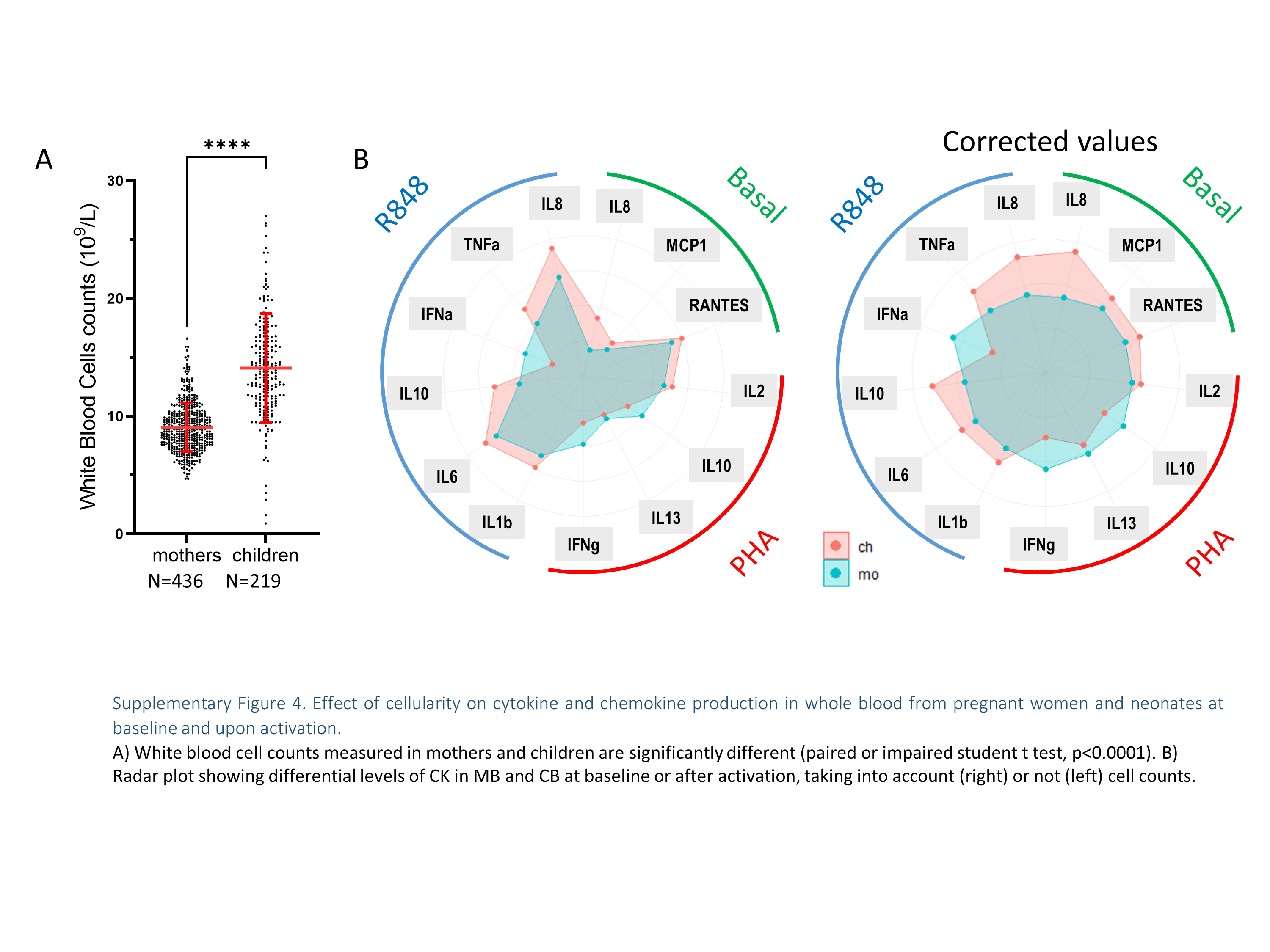

Supplement: Supplementary file 4 [file Image_4.tif]

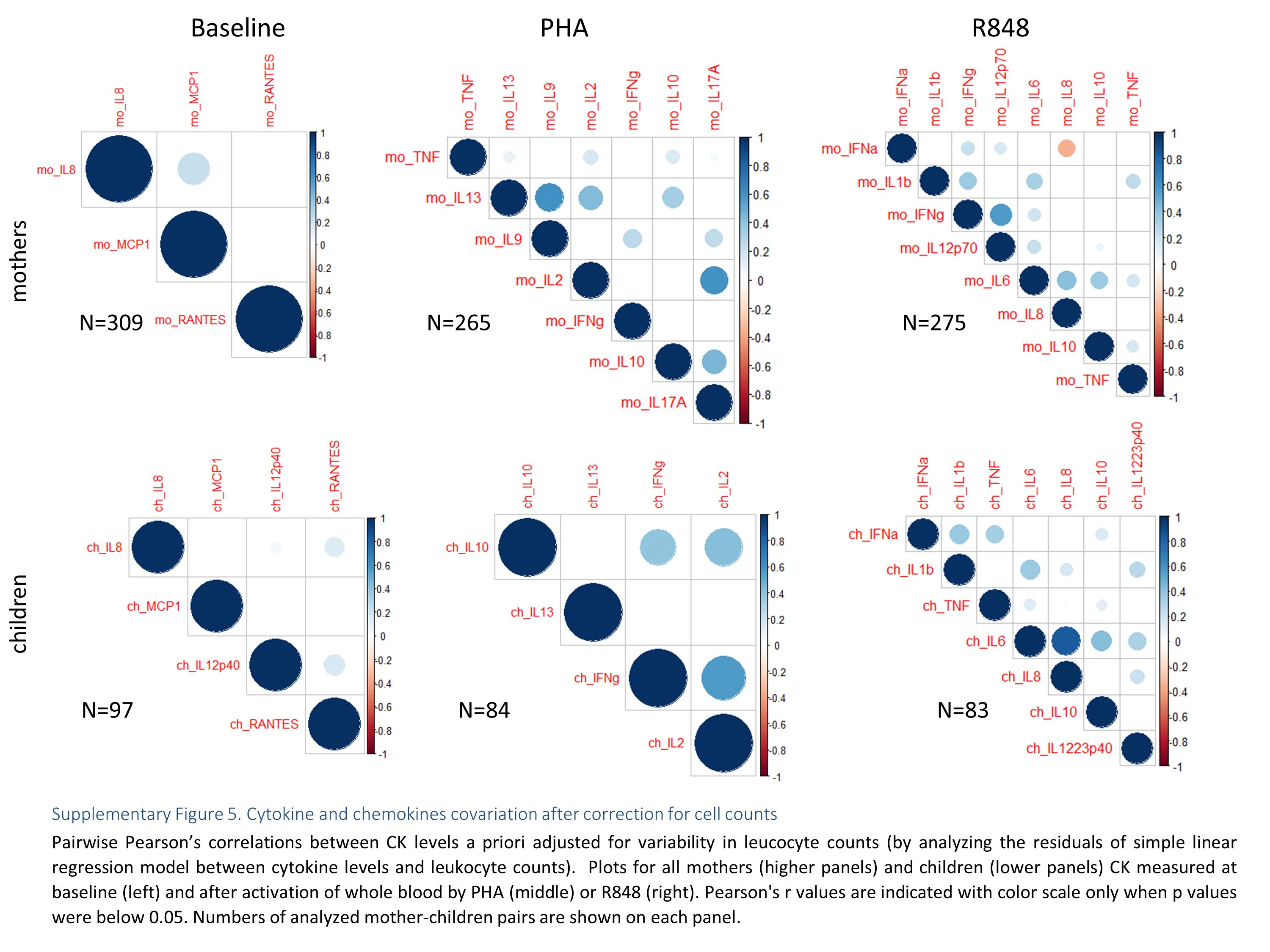

Supplement: Supplementary file 5 [file Image_5.tif]

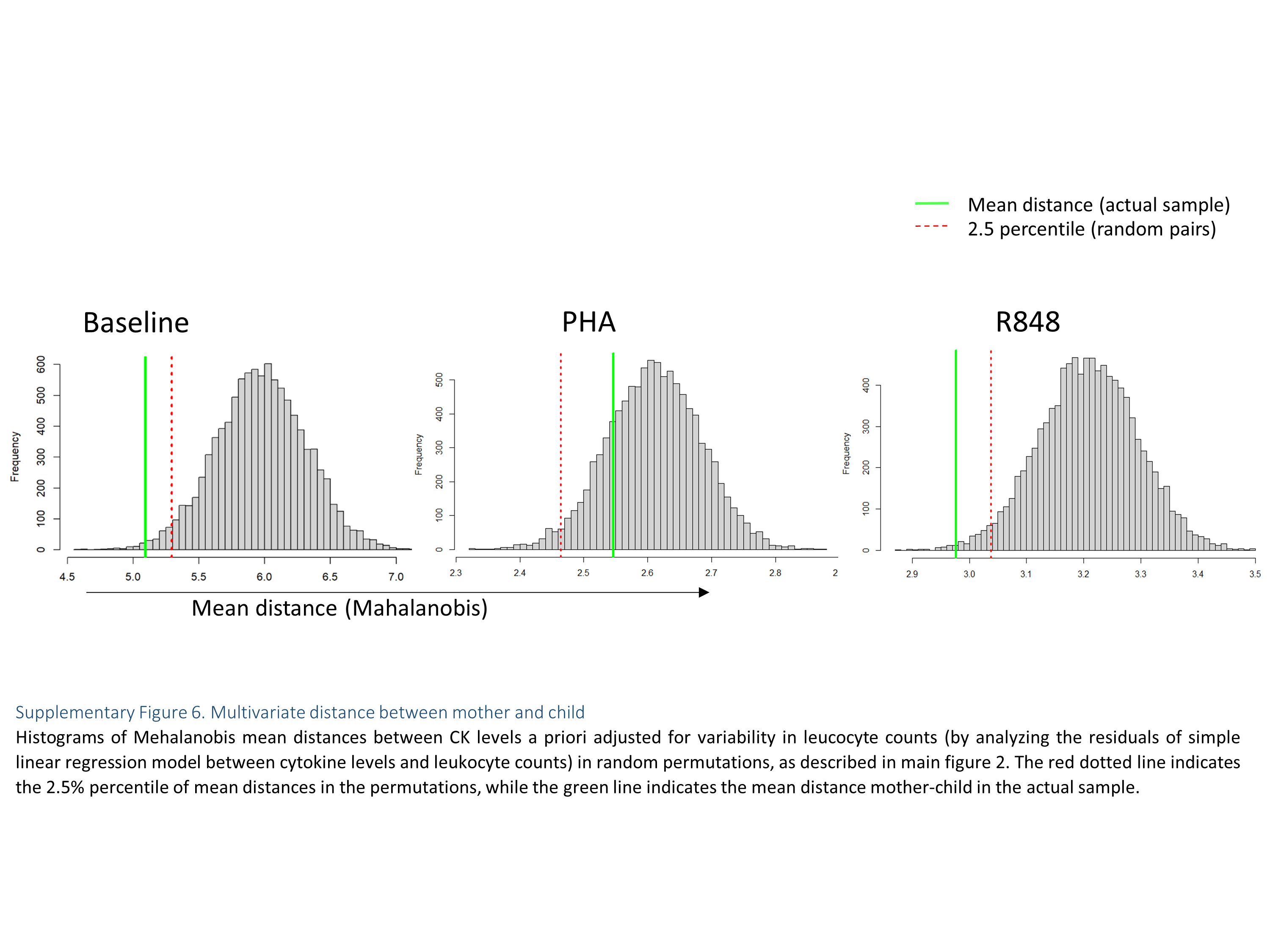

Supplement: Supplementary file 6 [file Image_6.tif]
